# Phytotherapeutic Intervention in Monosodium Glutamate-Induced Uterine Dysfunction: Efficacy of *Lepidium sativum, Prunus armeniaca, Stachys palustris,* and *Solenostemma argel*

**DOI:** 10.3390/ph19030521

**Published:** 2026-03-23

**Authors:** Eslam ElNebrisi, Nadia M. El Rouby, Fatimah Muaamar Noori, Nikoo Ali Jalali, Rodiana Mohamed Fouad Saber, Zainab Safieldin Abdalla Mohamed, Naglaa Gamil Shehab

**Affiliations:** 1 Basic Sciences Department, College of Medicine, Ras Al Khaimah Medical and Health Sciences University, Ras Al Khaimah 11172, United Arab Emirates; eslam@rakmhsu.ac.ae; 2 Department of Biomedical Sciences, Dubai Medical College for Girls, Dubai Medical University, Dubai 20170, United Arab Emirates; nadiah@dmu.ae; 3 College of Pharmacy, Dubai Medical University, Dubai 19099, United Arab Emirates; fmn20180207@dmu.ae (F.M.N.); cp2022006@dmu.ae (N.A.J.); or rodainawork12314@gmail.com (R.M.F.S.); zsam20180240@dmu.ae (Z.S.A.M.); 4 Pharmaceutical Sciences Department, College of Pharmacy, Dubai Medical University, Dubai 19099, United Arab Emirates; 5 Pharmacognosy Department, Faculty of Pharmacy, Cairo University, Cairo 11562, Egypt

**Keywords:** *Lepidium sativum*, *Prunus armeniaca*, *Solenostemma argel*, *Stachys palustris*, monosodium glutamate, uterine dysfunction

## Abstract

**Introduction**: Uterine fibroids are benign tumors arising from uterine smooth muscle and significantly affect women’s health worldwide. While conventional treatments often involve hormonal therapies or invasive surgeries, these approaches are limited by cost, side effects, and fertility concerns. This study aimed to evaluate the in vivo bioactivity of four medicinal plant extracts, *Lepidium sativum*, *Prunus armeniaca*, *Solenostemma argel,* and *Stachys palustris*, in ameliorating monosodium glutamate (MSG)-induced uterine changes in rats, providing preliminary preclinical evidence. **Methods**: The extracts were evaluated for their flavonoid and total phenolic contents, antioxidant capacity, and hormonal modulatory effects. Female Wistar rats were treated with monosodium glutamate to induce uterine changes, followed by interventions with herbal extracts. Outcomes were evaluated via biochemical, hormonal, and histological analyses. **Results**: Among the four extracts, *Lepidium sativum* and *Stachys palustris* showed superior antioxidant activity, restoring catalase, glutathione, and superoxide dismutase levels. These extracts also significantly reduced estrogen levels and estrogen receptor expression, correlating with improved histological outcomes, including reduced endometrial hyperplasia and myometrial thickness. *Solenostemma argel* and *Prunus armeniaca* exhibited moderate effects. **Conclusions**: This study underscores the potential of *Lepidium sativum* and *Stachys palustris* as natural therapeutic agents for fibroid management through antioxidant activity and hormonal modulation. Future research should focus on clinical validation to translate these findings into effective treatments.

## 1. Introduction

Uterine fibroids (UFs), also known as leiomyomas, are benign monoclonal tumors of smooth muscle origin that commonly affect women of reproductive age [1]. These tumors can present as asymptomatic or with symptoms such as heavy menstrual bleeding, pelvic pressure, pain, urinary frequency, and infertility. Globally, fibroids affect an estimated 70–80% of women by the age of 50, representing a significant public health concern due to their impact on quality of life, productivity, and healthcare costs. Despite their benign nature, fibroids are a leading cause of hysterectomies, raising concerns about the preservation of fertility, particularly in women who wish to conceive [2]. Monosodium glutamate (MSG) is known to elevate systemic oxidative stress, increase estrogen levels, and cause uterine hypertrophy—hallmarks of fibroid pathology [3,4,5].

The etiology of fibroids is multifactorial, with oxidative stress and hormonal imbalances, particularly hyperestrogenism, playing critical roles in their development and progression. Oxidative stress, driven by an imbalance between reactive oxygen species (ROS) and the body’s antioxidant defense, has been implicated in cellular proliferation, extracellular matrix (ECM) deposition, and smooth muscle hypertrophy within uterine tissue [6]. Similarly, estrogen promotes fibroid growth by stimulating ECM production and increasing vascular permeability, leading to the enlargement and persistence of these tumors. These mechanisms underline the need for treatments targeting both oxidative stress and hormonal pathways [6,7].

Current therapeutic options for uterine fibroids include hormonal therapies, such as gonadotropin-releasing hormone (GnRH) analogs, selective progesterone receptor modulators (SPRMs), and intrauterine devices, as well as non-hormonal options like non-steroidal anti-inflammatory drugs (NSAIDs) and antifibrinolytics [8,9]. While effective in symptom management, these treatments are associated with significant limitations, including high costs, side effects, and the inability to preserve fertility in many cases. Surgical interventions, such as myomectomy and hysterectomy, remain the standard for severe cases but are invasive and may not be acceptable to women desiring future pregnancies [8].

The limitations of conventional treatments have driven interest in complementary and alternative medicine, particularly the use of herbal remedies. Medicinal plants offer a natural, cost-effective, and fertility-preserving alternative, with many exhibiting antioxidant, anti-inflammatory, and hormonal regulatory properties. Medicinal plants have long been valued for their therapeutic properties, including antioxidant and anti-inflammatory effects, which are critical in addressing oxidative stress and hormonal imbalances implicated in uterine fibroids. In this study, the selection of *Lepidium sativum* (Garden cress), *Prunus armeniaca* (Apricot), *Stachys palustris* (Marsh woundwort), and *Solenostemma argel* (Argel) was guided by a combination of ethnopharmacological relevance and emerging biomedical evidence, particularly concerning their potential therapeutic effects on uterine changes [10,11]. Specifically, *Lepidium sativum* has been traditionally used in Middle Eastern and North African cultures to support female reproductive health, and *Stachys palustris* has been cited in European folk medicine for alleviating menstrual and uterine disorders [12]. These traditional uses suggest potential applications in managing uterine pathologies, including fibroid-like conditions. Coupled with reported antioxidant and hormone-modulating effects, these plants were selected to investigate their therapeutic role in MSG-induced uterine alterations.

*Lepidium sativum* (Garden cress), belonging to the family Brassicaceae, has been traditionally used to support reproductive health. Recent studies have demonstrated its ability to modulate gonadotropin secretion and improve reproductive outcomes in animal models, indicating its potential in addressing hormonal imbalances associated with uterine fibroids [10,13]. Its seed oil, rich in α-linolenic acid, has shown potential in protecting against oxidative damage.

*Prunus armeniaca* (Rosaceae family) is rich in bioactive compounds such as β-carotene and amygdalin. These constituents have been associated with antioxidant and anti-inflammatory properties, which are beneficial in mitigating oxidative stress and inflammation linked to fibroid pathogenesis [14].

*Stachys palustris*, (Marsh woundwort), a member of the Lamiaceae family, has a history of use in traditional medicine for its anti-inflammatory properties. Contemporary research supports these uses, highlighting its potential in alleviating symptoms related to uterine fibroids [15].

*Solenostemma argel* (Asclepiadaceae family), has been employed in traditional North African medicine for gynecological ailments. Scientific investigations have confirmed its uterine relaxant and anti-inflammatory activities, suggesting its utility in managing fibroid-related symptoms [16].

Collectively, these plants were selected based on their traditional use in reproductive health and scientific evidence supporting their roles in modulating oxidative stress, inflammation, and hormonal activity—key factors in the development and progression of uterine fibroids. This study specifically evaluated whether these plant extracts could reverse monosodium glutamate (MSG)-induced hyperestrogenism, oxidative imbalance, and uterine tissue alterations [10,17,18]. This study aimed to evaluate the in vivo bioactivity of four medicinal plant extracts in ameliorating monosodium glutamate (MSG)-induced uterine changes in rats, providing preliminary preclinical evidence to inform future clinical evaluation. Monosodium glutamate (MSG), widely used as a flavor enhancer, is known to induce oxidative stress and hormonal imbalances, making it a suitable model for studying uterine changes. Specifically, the study investigates the antioxidant activity, hormonal modulation, and histological effects of *Lepidium sativum*, *Stachys palustris*, *Solenostemma argel*, and *Prunus armeniaca*, providing insights into their potential as natural treatments for fibroids. By addressing both oxidative and hormonal pathways, this research fills a critical gap in the search for non-invasive, cost-effective, and fertility-preserving alternatives to current fibroid treatments.

## 2. Results

Statistical differences among groups were assessed by one-way ANOVA with Tukey’s multiple comparison test.

### 2.1. Phytochemical Investigation

#### 2.1.1. Extraction Yields

Extraction with absolute ethanol—followed by filtration and rotary evaporation of the filtrate at 50 °C—yielded variable amounts of residue from each plant. Among the tested extracts, *Solenostemma argel* demonstrated the highest residue percentage (18.2%), followed by *Stachys palustris* (7.1%). *Lepidium sativum* and *Prunus armeniaca* yielded lower percentages (5.0% each).

#### 2.1.2. Standardization of Plant Extracts

Standardization of plant extracts using TLC, investigation of total phenolic and flavonoid contents, and measurement of in vitro antioxidant activity ensure consistency, quality, and reproducibility of bioactive compounds.

This process is essential for reliable interpretation of biological study results and for comparing data across experiments.

#### 2.1.3. TLC Analysis

TLC analysis confirmed the presence of flavonoids and terpenes in all extracts. Key flavonoids identified include hesperidin in *Lepidium sativum* and *Prunus armeniaca* (Rf = 0.74) and apigenin in *Stachys palustris* (Rf = 0.82), while quercetin was detected only in *Solenostemma argel*. Similarly, terpenes—such as linoleic and oleic acids—were found predominantly in *Solenostemma argel* and *Prunus armeniaca*.

#### 2.1.4. Total Phenolic and Flavonoid Contents

Spectrophotometric analysis revealed that *Solenostemma argel* showed the highest flavonoid content (0.37 mg quercetin equivalent/g), followed by *Lepidium sativum*, *Prunus armeniaca,* and *Stachys palustris* (0.20, 0.17, and 0.13 mg quercetin equivalent/g, respectively), while *Stachys palustris* exhibited the highest phenolic content (0.75 mg gallic acid equivalent/g), followed by *Prunus armeniaca*, *Lepidium sativum*, and *Solenostemma argel* (0.59, 0.30, and 0.17 mg gallic acid equivalent/g, respectively).

#### 2.1.5. Antioxidant Activity

The antioxidant potential of the extracts was evaluated using the 2,2-diphenyl-1-picrylhydrazyl (DPPH) radical scavenging assay. *Solenostemma argel* and *Stachys palustris* exhibited the highest radical scavenging activity (92.26% and 92.47%, respectively), comparable to ascorbic acid (95.00%). *Lepidium sativum* and *Prunus armeniaca* showed slightly lower activity (90.24% and 83.94%, respectively).

### 2.2. Biological Study

#### 2.2.1. LD_50_

The choice of a single dose of 500 mg/kg for all plant extracts was based on previously published studies that demonstrated significant pharmacological activity at this concentration without inducing toxicity. This standardized dose allowed for a direct comparative evaluation of the extracts under uniform conditions [13,19,20,21].

#### 2.2.2. Antioxidant Enzyme Levels

The selection of biochemical markers in this study was guided by their mechanistic relevance to fibroid pathogenesis, specifically their involvement in oxidative stress and hormonal imbalance. Catalase (CAT) and superoxide dismutase (SOD) are key enzymatic antioxidants responsible for neutralizing reactive oxygen species (ROS), which are known to promote smooth muscle proliferation and extracellular matrix deposition in uterine fibroids. Glutathione (GSH), a major intracellular non-enzymatic antioxidant, plays a crucial role in maintaining redox balance, and its depletion is frequently observed in fibroid-affected tissues, contributing to cellular damage and inflammation. Estrogen (E2) is a well-established driver of fibroid growth, enhancing uterine vascularization and cellular proliferation, while estrogen receptor alpha (ER-α) mediates estrogenic signaling and is often upregulated in fibroid tissue, contributing to hormone sensitivity and lesion persistence. Similar biomarker panels have been used in fibroid and uterine remodeling studies to assess the effects of antioxidant and estrogen-modulating treatments [6,7]. All hormonal and biochemical ELISA assays were performed on rat serum samples.

#### 2.2.3. Catalase (CAT) Activity

**Figure 1:**
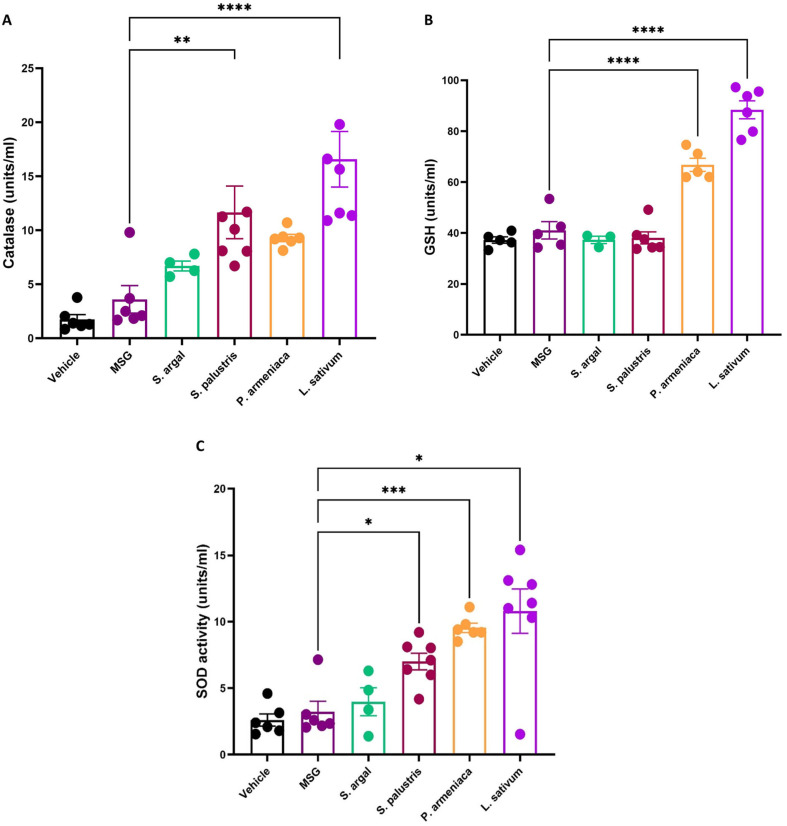
Effect of different herbal extracts on antioxidant enzyme activities in MSG-induced uterine changes in Wistar rats. (**A**) Catalase (CAT) activity. Treatment with *Lepidium sativum* significantly increased CAT activity compared to MSG (*p* < 0.0001), followed by *Stachys palustris* (** *p* < 0.01). (**B**) Glutathione (GSH) levels. *Lepidium sativum* and *Prunus armeniaca* significantly elevated GSH levels compared to MSG (**** *p* < 0.0001), with *L. sativum* showing the greatest effect. (**C**) Superoxide dismutase (SOD) activity. *Lepidium sativum* and *Prunus armeniaca* significantly improved SOD activity (*** *p* < 0.001 and * *p* < 0.05, respectively), while other extracts showed moderate increases. Data are expressed as mean ± SD (*n* = 6 per group). Statistical analysis was performed using one-way ANOVA followed by Tukey’s post hoc test. A *p*-value < 0.05 was considered statistically significant.

Treatment with *Lepidium sativum* significantly enhanced catalase activity, demonstrating the most pronounced effect among the tested plant extracts (*p* < 0.0001 ****). *Stachys palustris* also resulted in a statistically significant increase in catalase levels (*p* < 0.01 **), although to a lesser extent. In contrast, *Solennostemma argel* and *P. armeniaca* showed modest increases in catalase activity compared to the MSG group. These findings suggest that *Lepidium sativum* and *Stachys palustris* are particularly effective in enhancing antioxidant enzyme activity, potentially offering protection against MSG-induced oxidative stress (Figure 1A).

#### 2.2.4. Glutathione (GSH) Levels

Treatment with herbal extracts significantly improved GSH levels, with *Lepidium sativum and P. armeniaca* demonstrating the most pronounced effect (*p* < 0.0001 ****), further supporting their antioxidant potential. These findings emphasize the protective effects of the tested plant extracts against MSG-induced oxidative damage. The comparative results are illustrated in Figure 1B.

#### 2.2.5. Superoxide Dismutase (SOD) Activity

Treatment with herbal extracts led to a notable enhancement of SOD activity in rats exposed to MSG. Among the tested groups, *Lepidium sativum* demonstrated the highest increase in mean SOD levels; however, due to greater variability among samples, the improvement was statistically modest (*p* < 0.05). In contrast, *Prunus armeniaca* produced a slightly lower SOD increase but with tighter data distribution, resulting in a more statistically significant effect (*p* < 0.001 ***). *Stachys palustris* and *Solennostemma argel* showed significant elevations in SOD activity as well (*p* < 0.05). The comparative efficacy of the treatments is illustrated in Figure 1C.

### 2.3. Hormonal Analysis

**Figure 2:**
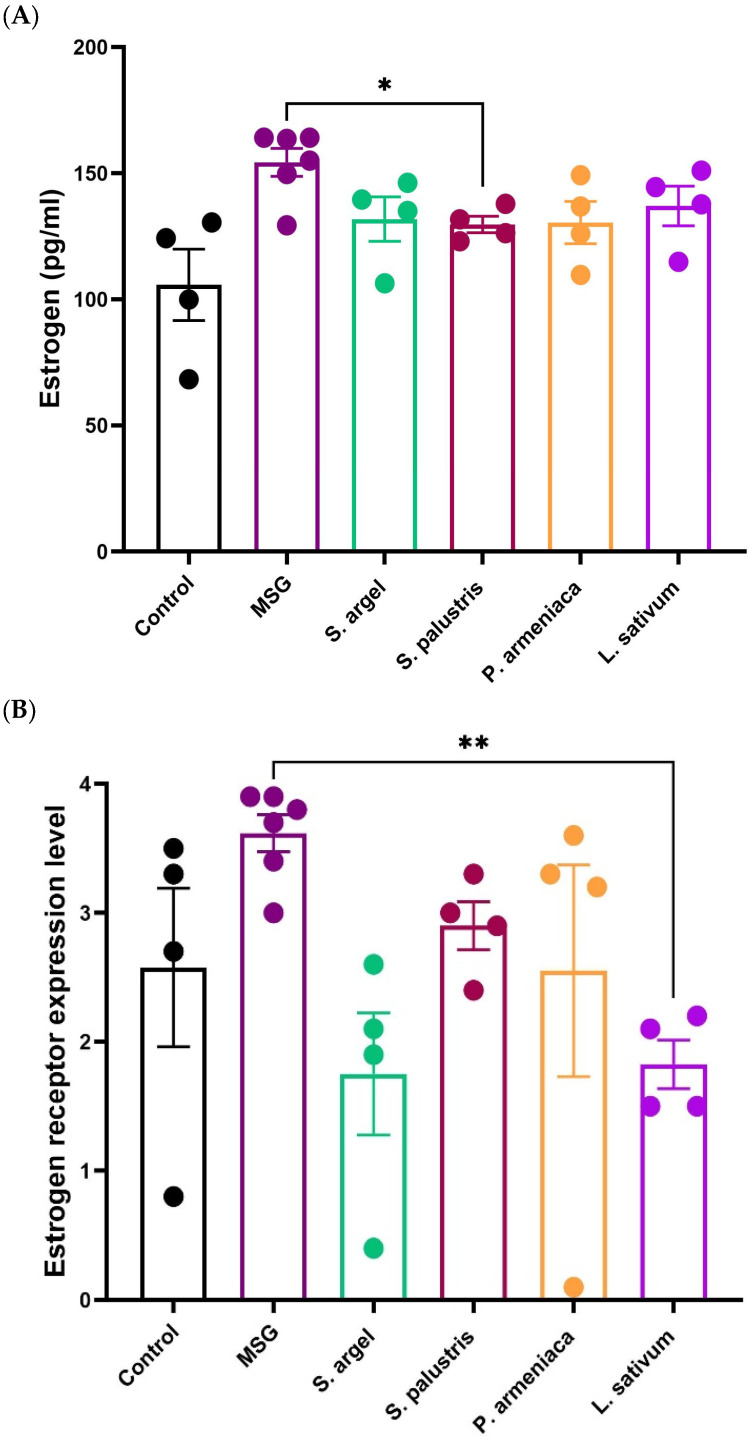
Effect of different herbal extracts on serum estradiol levels and uterine estrogen receptor alpha (ER-α) expression in MSG-induced uterine changes in Wistar rats. (**A**) Serum 17β-estradiol (E2) concentrations. MSG administration significantly increased estradiol levels compared to control. Treatment with *Stachys palustris* significantly reduced estradiol levels compared to MSG (* *p* < 0.05), while other extracts showed a non-significant decreasing trend. (**B**) Uterine ER-α expression levels. ER-α levels were significantly reduced in the *Solenostemma argel* and *Lepidium sativum* groups compared to MSG (** *p* < 0.01). No significant difference was observed between MSG and control. Data are presented as individual values (*n* = 6 per group) with mean ± SD. Statistical analysis was performed using one-way ANOVA followed by Tukey’s post hoc test. A *p*-value < 0.05 was considered statistically significant.

MSG administration induced a significant elevation in serum 17β-estradiol levels, indicating a hyperestrogenic state associated with uterine pathological changes. The MSG group recorded an average estradiol concentration of 154.3 ± 13.6 pg/mL, higher than the control group (105.7 ± 28.2 pg/mL). Among the treatment groups, *Stachys palustris* significantly reduced estrogen levels compared to MSG (*p* < 0.05), bringing the mean concentration down to 129.7 ± 6.5 pg/mL. *Solenostemma argel* (131.8 ± 17.6 pg/mL), *Prunus armeniaca* (130.4 ± 16.8 pg/mL), and *Lepidium sativum* (137.0 ± 15.7 pg/mL) also showed a trend toward reduction, but these changes were not statistically significant. Overall, the data suggest that *Stachys palustris* exerted a more prominent estrogen-modulating effect, with other extracts showing partial improvements (Figure 2A and Table 1).

MSG administration led to an increase in uterine ER-α expression compared to the control group, although this difference was not statistically significant. However, treatment with *Lepidium sativum* significantly reduced ER-α expression levels relative to MSG (*p* < 0.01), suggesting a potential estrogen receptor-modulating effect. *Solenostemma argel*, *Stachys palustris,* and *Prunus armeniaca* showed numerically lower ER-α levels than MSG, but these reductions were not statistically significant. These findings support the selective ability of certain extracts—particularly *L. sativum*—to attenuate MSG-induced upregulation of estrogen receptor expression in uterine tissue (Figure 2B).

### 2.4. Histological Results

**Figure 3:**
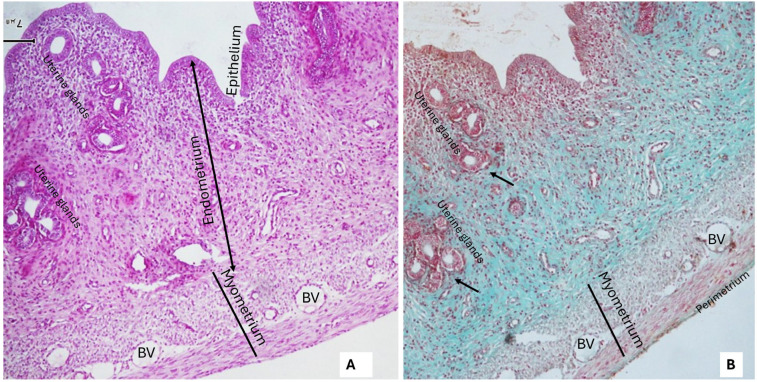
Photomicrographs of the uterus of a rat from the group that received distilled water only (control group). (**A**): Showing normal uterine architecture with intact endometrial epithelium and uterine glands. The uterine layers (endometrium, myometrium, and perimetrium) appear normal (H&E ×100). (**B**): There is mild deposition of collagen fibers in the endometrial stroma, around uterine glands (black arrows) and uterine blood vessels (BV) (thin black arrows) (Masson’s trichrome ×100).

The uteri of control rats (received distilled water, DW, only) showed normal uterine microstructure in the form of normal intact uterine layers (Endometrium, myometrium, and perimetrium). The endometrium showed normal uterine glands. Both the endometrium and its glands were lined with simple columnar partially ciliated and partially secretory epithelium. Using Masson’s trichrome stain, there was mild deposition of collagen fibers around uterine glands and blood vessels (Figure 3A,B).

**Figure 4:**
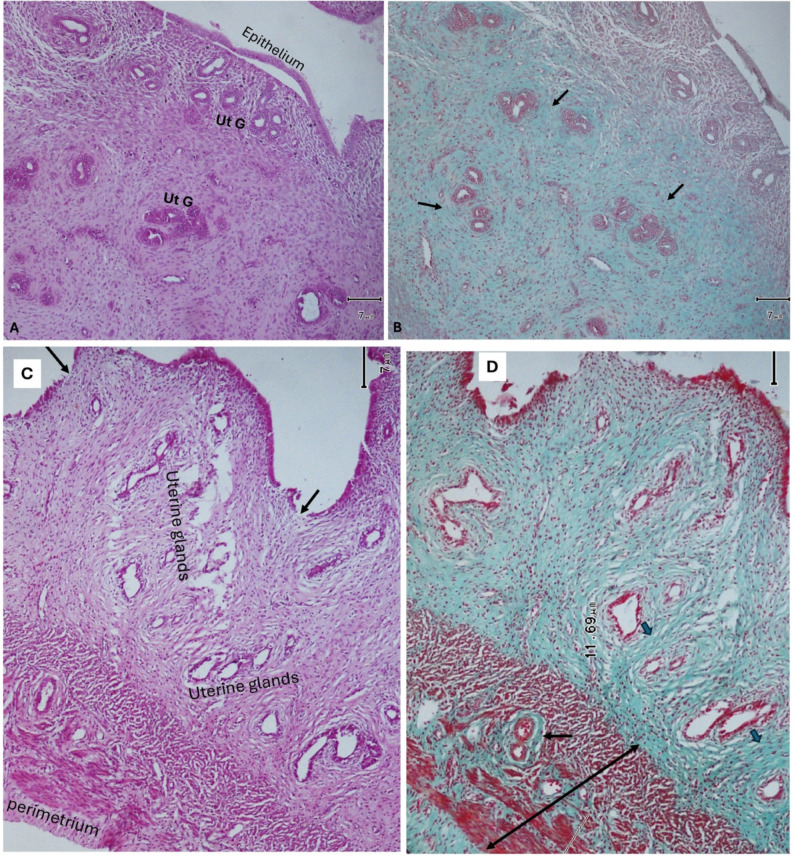

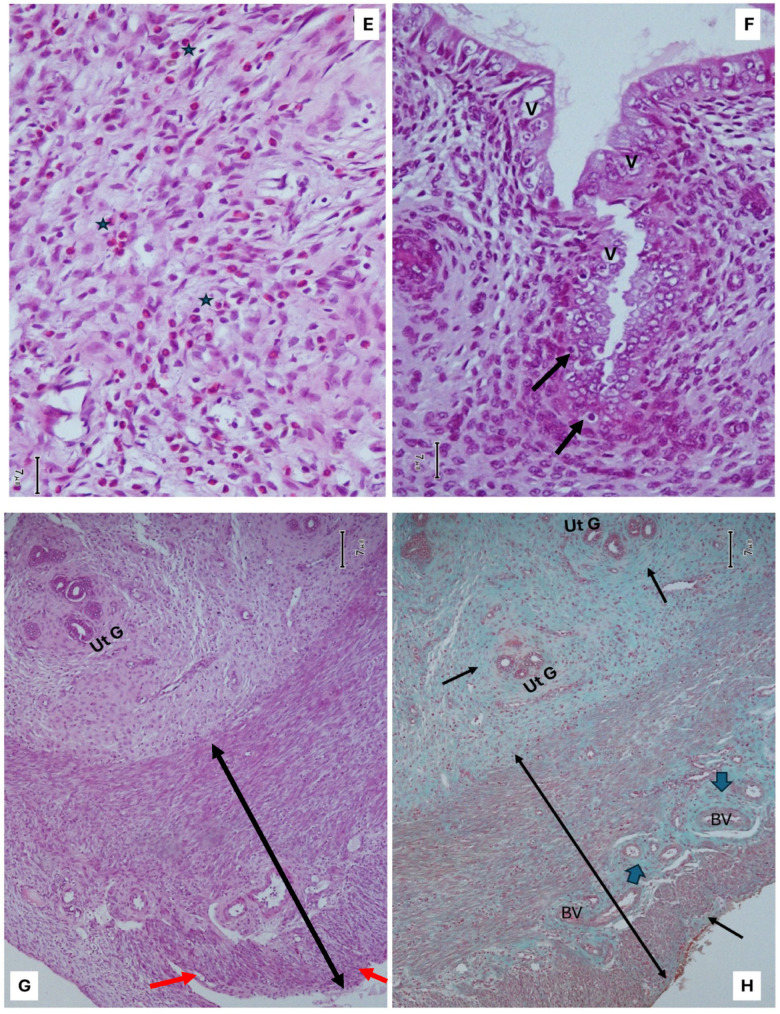
Uterine sections from rats that received MSG only, (**A**): reveal many uterine glands (Ut. G) and detached endometrial epithelium (H&E ×100). (**B**): A Masson’s trichrome (×100)-stained section shows moderate deposition of collagen fibers around uterine glands (arrows). (**C**,**D**): The sections reveal damaged endometrial epithelium (black arrows) with dilated distorted uterine glands. The myometrium (double headed arrow) shows thickening. There is a marked amount of collagen fibers deposited around uterine glands (blue arrows) and blood vessels (black arrows) (H&E and Masson’s trichrome ×100, respectively). (**E**,**F**): H&E-stained sections (×400), (**E**): shows mononuclear cellular infiltration of endometrium (stars), mainly eosinophils. (**F**): reveals hypertrophy, hyperplasia, and vacuolar (V) degeneration of the endometrial epithelium. Some apoptotic epithelial cells are seen (arrows). (**G**): The uterine sections demonstrate marked thickening of the myometrium (double-headed arrow) and bulging on the surface between the two red arrows (H&E ×100). (**H**): Masson’s trichrome-stained section (×100) shows deposition of a marked amount of collagen fibers around uterine glands (Ut. glands) (thin arrows). The uterine blood vessels (BV) seen between the layers of myometrium are surrounded with a heavy amount of collagen fibers (thick arrows).

In rats that received Monosodium glutamate (MSG) only, the uteri showed marked changes in their histological architecture. Some uteri revealed detached endometrial epithelium. Others showed endometrial hypertrophy as well as hyperplasia and vacuolar degeneration of the epithelial lining. Some epithelial apoptotic cells were seen. There were hyperplasia, hypertrophy, and distorted uterine glands. Also, the thickened endometrium revealed mononuclear cellular infiltration mainly with eosinophils. The myometrium showed smooth muscle cell proliferation and hypertrophy associated with disarrangement and bulging on the surface. Masson’s trichrome stain demonstrated moderate to heavy collagen fiber deposition around uterine glands and blood vessels. The myometrium appeared more vascular (Figure 4A–H, Table 2, Table 3 and Table 4).

**Figure 5:**
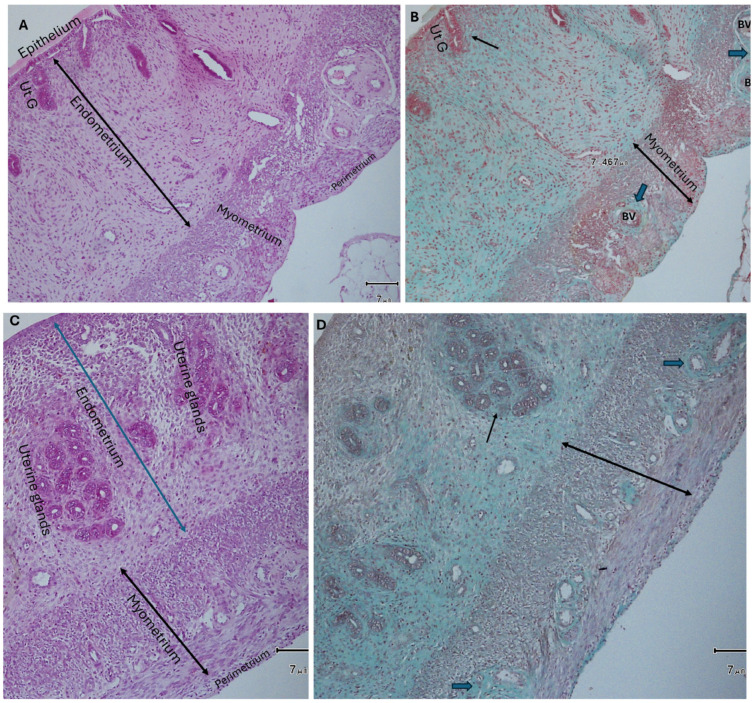
(**A**,**B**): Uterine sections from a rat treated with *Lepidium sativum* after MSG reveal marked improvement of the endometrium in the form of intact thin epithelium. There is a marked reduction in uterine glands (Ut. G). A mild amount of collagen fibers is deposited around uterine glands (Ut. G) (thin arrows), while a moderate amount of collagen (thick arrows) is deposited around the blood vessels (BV). The myometrium appears to have a normal thickness (H&E and Masson’s trichrome ×100, respectively). (**C**,**D**): Uterine sections of rats treated with *Stachys palustris* extract after MSG treatment revealed intact normal endometrial epithelium and normally structured hyperplastic uterine glands. The myometrium still shows some thickening but is more vascular. Mild deposition of collagen fibers was observed around uterine glands (thin arrow), but the uterine blood vessels appeared surrounded with a moderate amount of collagen fibers (thick arrow) (H&E and Masson’s trichrome ×100, respectively).

Uterine sections from rats treated with *Lepidium sativum* after monosodium glutamate revealed marked improvement of the endometrium in the form of intact thin epithelium. Also, there was a marked reduction in the number of uterine glands, which were lined with simple columnar epithelium. A mild amount of collagen fibers was deposited around uterine glands, while the blood vessels were surrounded with a moderate amount of collagen fibers. Also, a marked reduction of myometrium thickness was detected (Figure 5A,B). When female rats were treated with *Stachys palustris* extract after MSG, their uteri revealed intact normal endometrial epithelium. There was hyperplasia of normally structured uterine glands. The myometrium still showed mild thickening. A mild-to-moderate deposition of collagen fibers around uterine glands and blood vessels was observed (Figure 5C,D, Table 2, Table 3 and Table 4).

**Figure 6:**
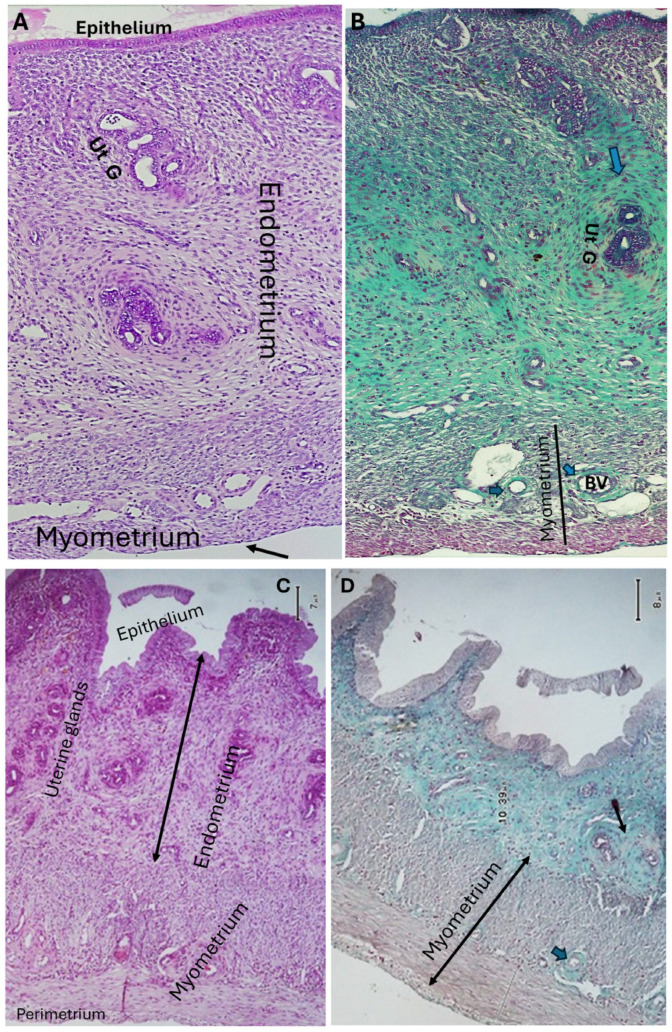
(**A**,**B**): Uterine sections of a rat given *Solennostemma argel* after MSG revealed a normal endometrium and uterine glands (Ut.G). There is moderate collagen fiber deposition around uterine glands and blood vessels (BV) (blue arrows). The myometrium appears normal (black line). The perimetrium is seen (black arrow) (H&E and Masson’s trichrome ×100, respectively). (**C**,**D**): Uterine sections of a rat received *Prunus armeniaca* after MSG show normal intact endometrial epithelium. The uterine glands are more numerous but of normal structure. The myometrium appears slightly thickened. There is a moderate amount of collagen fiber deposited around both uterine glands (black arrows) and blood vessels (blue arrows) (H&E and Masson’s trichrome ×100, respectively).

Administration of *Solennostemma argel* after monosodium glutamate to female rats revealed marked improvement of the endometrium. The uterine glands appeared normal in number and structure and were surrounded with a moderate amount of collagen fibers. The myometrium appeared normal, and its numerous blood vessels were surrounded with moderate amounts of collagen fibers (Figure 6A,B). When female rats received *Prunus armeniaca* after MSG, their uteri showed intact endometrial epithelium. The uterine glands were numerous but of normal structure. The myometrium appeared moderately thickened. There was a moderate amount of collagen fibers deposited around both uterine glands and blood vessels (Figure 6C,D, Table 2, Table 3 and Table 4).

Statistically, the MSG group exhibited a highly significant increase in collagen deposition as well as myometrial thickening compared to the control group (*p* < 0.001).

All treated groups with (*S. argal*, *P. armineana*, *L. Sativum*, and *S. palustris*) after MSG showed a statistically significant difference from the MSG group (*p* < 0.05). Data were analyzed using one-way AVOVA followed by Tukey’s post hoc test. A *p* < 0.05 was considered significant, as shown in Table 4.

## 3. Discussion

The etiology of uterine fibroids is multifactorial, with oxidative stress and hormonal imbalances, particularly hyperestrogenism, playing critical roles in their development and progression. Oxidative stress, driven by an imbalance between reactive oxygen species (ROS) and the body’s antioxidant defense, has been implicated in cellular proliferation, extracellular matrix (ECM) deposition, and smooth muscle hypertrophy within uterine tissue [6]. Similarly, estrogen promotes fibroid growth by stimulating ECM production and increasing vascular permeability, leading to the enlargement and persistence of these tumors. These mechanisms underline the need for treatments targeting both oxidative stress and hormonal pathways [6,7]. In this study, the selection of the four plants, *Lepidium sativum* (Garden cress), *Prunus armeniaca* (Apricot seed), *Stachys palustris* (Marsh woundwort), and *Solenostemma argel* (Argel) was guided by a combination of ethnopharmacological relevance and emerging biomedical evidence, particularly concerning their traditional medicinal uses and their potential antioxidant activity [10,11,12].

This study aimed to explore the in vivo bioactivity of four medicinal plant extracts, *Lepidium sativum*, *Prunus armeniaca*, *Solenostemma argel,* and *Stachys palustris*, in ameliorating monosodium glutamate (MSG)-induced uterine changes in rats, providing preliminary preclinical evidence. The four plant extracts were assessed for their antioxidant enzyme activities, hormonal regulatory effects, and histopathological impact on uterine tissue. Our findings demonstrate that MSG administration induced pathological uterine changes, characterized by increased oxidative stress, hyperestrogenism, elevated estrogen receptor alpha (ER-α) expression, and collagen accumulation. These observations are consistent with established literature linking estrogen dominance and oxidative imbalance to the development and progression of uterine changes [6,7].

Among the tested extracts, *Lepidium sativum* showed the most marked and consistent therapeutic effects. It significantly enhanced catalase (CAT), glutathione (GSH), and superoxide dismutase (SOD) levels, reflecting potent antioxidative capacity. Notably, *Lepidium sativum* also reduced ER-α expression, suggesting a dual mechanism involving the reduction of oxidative stress and modulation of the estrogen pathway. Although estradiol levels were not significantly reduced, the crude extract’s effect on ER expression may still contribute to a functional hormonal correction. Multiple studies have confirmed the potent antioxidant activity of *Lepidium sativum*, showing that its seed extracts significantly boost antioxidant enzyme levels (CAT, GSH, SOD) and reduce markers of oxidative stress, in agreement with the current findings [13,22,23]. These effects are consistent with previous studies that have reported the antioxidant activity of *Lepidium sativum* seeds, attributed to their high content of α-linolenic acid (an essential omega-3 fatty acid) and hesperidin, both of which can neutralize reactive oxygen species [18].

In addition, another study investigated the presence of ferulic and gallic acid [23].

The combination of flavonoids and phenolic acids with omega-3 fatty acids may act synergistically by enhancing antioxidant capacity and modulating inflammatory pathways, thereby contributing to the observed marked improvement in uterine parameters.

*Stachys palustris* also exhibited strong antioxidant properties, particularly enhancing catalase and SOD activity. Although it did not significantly reduce ER-α expression, it lowered estradiol levels more than any other extract. This selective hormonal action supports previous reports highlighting the phytoestrogenic and anti-inflammatory effects of phenolic compounds in *Stachys* species [15]. However, apigenin was detected in *S. palustris* via TLC in our analysis only; advanced phytochemical analyses, such as HPLC-ESI-MS and UPLC-PDA-ESI-TQD-MS/MS, have identified and quantified a variety of bioactive constituents in *S. palustris*, including flavones such as luteolin, which exhibited higher antioxidant activity [15,24].

*Prunus armeniaca* seeds exhibited moderate antioxidant effects (83.94%) and were statistically the most significant in enhancing SOD activity. This activity is likely due to their rich content of vitamin E (tocopherols) [25] and unsaturated fatty acids, dominated by oleic acid (approx. 29–65%) and linoleic acid (approx. 11–32%) with a lack of flavonoids [10,26,27]. While it did not reduce ER-α expression, it achieved a modest decrease in estradiol, supporting a mild estrogen-modulating potential. Previous research has also highlighted its tissue-protective, anti-inflammatory, and anticancer properties, attributed to these bioactive compounds [28,29].

*Solenostemma argel* exhibited comparatively modest antioxidant and hormonal effects relative to other extracts. Notably, its pronounced ability to downregulate ER-α expression suggests that its therapeutic action may be mediated through direct modulation of estrogen receptors rather than by broadly suppressing systemic estrogen levels [30]. Nevertheless, the observed variability in its influence on catalase and superoxide dismutase (SOD) activities points to inconsistencies likely stemming from the plant’s inherent chemical heterogeneity [31]. *Solenostemma argel* is a medicinal plant rich in bioactive compounds, primarily including pregnane glycosides, phenolic acids, flavonoids (e.g., kaempferol, quercetin), sterols, and triterpenoids, to which the antioxidant activities can be attributed [31].

Histologically, MSG-induced uterine changes mimicked fibroid-like pathology, including myometrial thickening, endometrial hyperplasia, glandular distortion, and marked collagen deposition. These findings resemble the fibroid-related ECM remodeling described by Deligdish and Loewenthal (1970) [25]. Treatments with *Lepidium sativum* and *Stachys palustris* reversed most histological abnormalities, demonstrating improved endometrial architecture and reduced collagen area percentage. These structural improvements correlate with biochemical outcomes, reinforcing their therapeutic relevance. Quantitative collagen deposition and myometrial thickening were analyzed using an image analyzer, which further validated the protective role of all four extracts, with *Lepidium sativum* exhibiting the most substantial effect.

Although MSG-induced uterine changes may not replicate all aspects of human fibroids, the model is widely used to simulate key pathogenic features, including oxidative stress, estrogen imbalance, and myometrial hypertrophy. Previous studies have utilized MSG to induce uterine hyperplasia and ECM remodeling, establishing its utility in preliminary fibroid-related investigations. These pathophysiological changes mirror several hallmarks of fibroid biology, validating its relevance as a preclinical model [32].

Some considerations should be noted in interpreting the findings. Although TLC and DPPH methods provided initial insight into the phytochemical and antioxidant profiles of the extracts, they are preliminary tools and could be complemented in future studies with more advanced methods such as LC-MS to ensure compound specificity and reproducibility. Additionally, while the MSG-induced model effectively reproduced oxidative and hormonal imbalances relevant to fibroid pathology, it may not fully capture the complexity of true uterine fibroids. Nevertheless, it remains a useful and widely accepted platform for evaluating early therapeutic responses. Finally, the use of commercially sourced plant materials may introduce variability, though efforts were made to standardize extraction and dosing across groups. Larger studies using cultivated and authenticated plant material could strengthen the reliability of the outcomes.

While this study focused on serum-based measurements of antioxidant enzymes and hormonal markers, it did not quantify their concentrations directly in uterine tissue. Future studies should analyze tissue-level concentrations of the key hormones and oxidative stress markers to validate systemic findings and better correlate with histological outcomes. In addition, although the MSG-induced rat model effectively reproduces key features of oxidative stress and hyperestrogenism, translational extrapolation to human fibroid pathology requires careful consideration. The rat estrous cycle (4–5 days) differs substantially from the human menstrual cycle (≈28 days), both in duration and hormonal dynamics. In the present study, estrous cycle phase was not synchronized prior to sacrifice, which may have contributed to hormonal variability. Future investigations should incorporate estrous cycle monitoring and evaluate cyclic or long-term treatment regimens to better mimic human reproductive physiology. Moreover, clinical translation would require dose optimization, safety profiling, and assessment across menstrual phases to determine sustained therapeutic benefit.

In conclusion, this study provides compelling preclinical evidence that *Lepidium sativum* and *Stachys palustris* possess therapeutic potential against fibroid-like uterine pathology through their combined antioxidant and estrogen-modulating effects. While *Prunus armeniaca* and *Solenostemma argel* showed milder effects, their distinct receptor or antioxidant actions warrant further mechanistic investigation. The findings lay the groundwork for future in vivo and clinical studies exploring these extracts as fertility-preserving, plant-based alternatives in fibroid management.

## 4. Materials and Methods

### 4.1. Chemicals

All chemicals used in this study were of analytical grade. Absolute ethanol, methanol, chloroform, ethyl acetate, benzene, sodium nitrate, aluminum chloride, sodium hydroxide, sodium carbonate, and carboxymethylcellulose (CMC) were purchased from Fisher Scientific Company. Monosodium glutamate (MSG) and 2,2-diphenyl-1-picrylhydrazyl (DPPH) were obtained from Sigma Chemicals Co. (St. Louis, MO, USA). The Folin-Ciocalteu reagent was procured from Merck (Darmstadt, Germany).

### 4.2. Plant Material and Extraction

The seeds of *Lepidium sativum* and *Prunus armeniaca*, (500 g each) the leaves of *Solenostemma argel* (492 g) and the whole plant of *Stachys palustris* (350 g), were sourced from the local market in the UAE in September 2021. Plant authentication was conducted by Prof. Naglaa Shehab, Department of Pharmaceutical Sciences, College of Pharmacy, Dubai Medical University, Dubai, UAE. Voucher specimens were deposited at the Herbarium of the Pharmaceutical Sciences Department (reference #6–10–21).

The plants were air-dried at room temperature and then ground into fine powder. The powdered materials were extracted by cold maceration in absolute ethanol (5 L × 2 each) for a period of 14 days to ensure maximum extraction of bioactive compounds. The solvent in each case was evaporated under reduced pressure at 50 °C using a rotary evaporator, and the resulting residues were stored at 4 °C in airtight containers until further use.

### 4.3. Phytochemical Investigation

Standardization of plant extracts.

#### 4.3.1. Thin Layer Chromatography (TLC)

The presence of flavonoids and terpenes in the plant extracts was analyzed using TLC plates. For flavonoids, a solvent system of methanol and chloroform in an 8:2 ratio was employed, while terpenes were separated using a benzene and ethyl acetate system in an 8.2:1.4 ratio. The spots were detected and visualized under a UV lamp at 365 nm, both before and after exposure to ammonia vapors and p-anisaldehyde solution at 110 °C.

#### 4.3.2. Total Phenolic and Flavonoid Contents

The total phenolic and flavonoid contents were estimated via a spectrophotometer (UV-1800 PharmaSpec, Shimadzu, Japan). All the experiments were carried out in triplicate. The total phenolic contents were determined by using the Folin-Ciocalteu reagent as described by Singleton and Rossi [20] and modified by Oktay et al. [19]. Results were shown as mg/g gallic acid equivalent, calculated on the dry weight of plant material; serial dilutions of gallic acid (10, 20, 30, 40, and 50 μg/mL) were used for the establishment of the calibration curve. Aliquots of 1 mL of each plant extract was added to a volumetric flask containing 9 mL of distilled water and 1 mL of Folin-Ciocalteu reagent and the reaction mixture was carefully mixed. After 5 min, 10 mL of 7% sodium bicarbonate was added to the mixture, after which it was incubated in the dark at room temperature for 90 min. Finally, the absorbance of the aliquots was measured at 750 nm against the reagent blank.

The total flavonoid content of the plant extracts was determined spectrophotometrically by the aluminum chloride method as described by Dewanto et al. [33]. In total, 1 mL of each plant extract was added to 3 mL of distilled water and 0.3 mL of 5% sodium nitrite. After 5 min, 0.3 mL of 10% aluminum chloride was then added, and the mixture was left for 5 min. Finally, 2 mL of 1 mM sodium hydroxide and 10 mL of distilled water were added. The absorbance of the mixture was then measured at 510 nm in the spectrophotometer.

#### 4.3.3. DPPH Radical Scavenging Assay

The free radical scavenging activity of the extracts was assessed using the 2,2-diphenyl-1-picrylhydrazyl (DPPH) assay. Extracts (200 mg) were dissolved in 20 mL of 70% ethanol. For each sample, 2 mL of the extract solution was mixed with 2 mL of 0.1 mM DPPH solution in ethanol. After 30 min of incubation in the dark at room temperature, the absorbance was measured at 517 nm using a UV–vis spectrophotometer. The percentage inhibition of DPPH radicals was calculated as follows:

% inhibition = [A_0_ − (A_1_ − A_2_)]/A_0_ × 100%

where A_0_ is the absorbance of the control (ethanol and DPPH), A_1_ is the absorbance in the sample in ethanolic DPPH, and A_2_ is the absorbance of the sample with ethanol. Samples were analyzed in triplicate and compared using ascorbic acid as positive control.

### 4.4. Biological Study

Acute toxicity studies of all four plants have been previously published [21,34,35].

#### 4.4.1. Animal Model

Ethical approval for this study was obtained from the Dubai Pharmacy College for Girls Ethics Committee (REC/UG/2021/06, approval date: 9 November 2021). All animal procedures were conducted in accordance with the guidelines of the National Society for Medical Research and the National Institutes of Health’s Guide for the Care and Use of Laboratory Animals and as per the ethical standards for laboratory experimental animals.

A total of forty-six healthy, non-pregnant female Wistar rats (weighing 150 ± 15 g) were procured from the in-house breeding facility at the Animal House of the College of Pharmacy, Dubai Medical University, Dubai, UAE. Animals were acclimatized for 14 days prior to the experiment. They were housed in individually ventilated polycarbonate cages (dimensions: 40 × 25 × 20 cm) with sterilized corn cob bedding, which was replaced every 2–3 days. The animals were maintained under controlled conditions: 12 h light/dark cycle, an ambient temperature of 23 ± 2 °C, and relative humidity of 55–60%. Standard rodent chow pellets (commercially available from [Fujairah Feed Factory, Fujairah, UAE; http://www.fujairahfeed.ae/rodent-feed.html (accessed on 17 March 2026)]) were provided ad libitum, and animals had free access to filtered water throughout the study.

Biological sample collection was performed under light ether anesthesia. Blood was obtained via cardiac puncture, and uterine tissues were excised post-mortem, rinsed in cold saline, and either fixed in 10% formalin for histological analysis or snap-frozen and stored at –80 °C for biochemical assays.

Power Analysis: A priori power analysis was conducted using G*Power version 3.1.9.7 to determine the minimum number of animals required per group. Based on preliminary data and assuming a 20% effect size in antioxidant enzyme levels (e.g., catalase activity), with a power of 80% (1 − β = 0.80) and a significance level of α = 0.05, the required sample size was calculated to be 6 animals per group. To account for possible biological variability and attrition, some groups included 7 animals. The experimental unit was a single individual rat.

#### 4.4.2. Experimental Design

Biochemical and statistical analyses were conducted with investigators blinded to group allocation. Rats were randomly divided into seven groups (*n* = 6–7 per group) (Table 1):

- Control: Received distilled water via oral gavage.
- Monosodium glutamate (MSG)-treated: Received MSG (900 mg/kg) + distilled water via oral gavage.
- *Solennostemma argel* group (MSG) 900 mg/kg (induction) + 500 mg/kg extract in CMC via oral gavage. (treatment).
- *Stachys palustris* group (MSG) 900 mg/kg (induction) + 500 mg/kg extract in CMC via oral gavage (treatment)
- *Prunus armeniaca* group (MSG) 900 mg/kg (induction) + 500 mg/kg extract in CMC via oral gavage (treatment).
- *Lepidium sativum* group (MSG) 900 mg/kg (induction) + 500 mg/kg extract in CMC via oral gavage (treatment)

The doses of the plant extracts (500 mg/kg) were selected based on previously published LD_50_ values, ensuring safety and biological activity. The LD_50_ of *Solennostemma argel*, *Lepidium sativum,* and *Stachys palustris* is relatively high, suggesting low toxicity (up to 5000 mg/kg). The LD_50_ of *Prunus armeniaca* was found to be 2000 mg/kg. Prior toxicological studies support this dose range as non-toxic and effective in eliciting physiological response [7,13,21,23,24].

**Figure 7:**
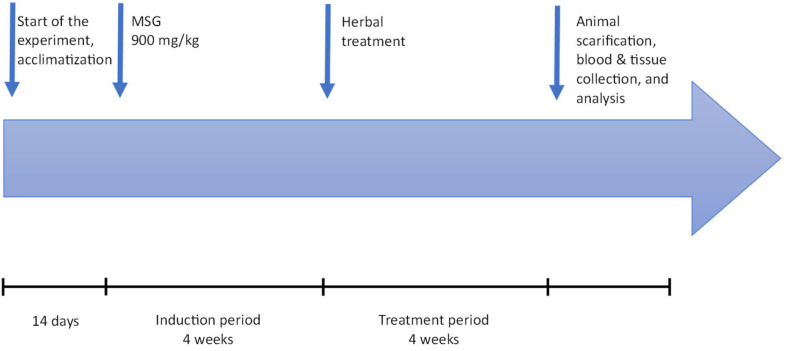
For the induction of uterine fibroids, MSG was used at the dose of 900 mg/kg, and the animals of groups II, III, IV, V, and VI received 900 mg/kg of MSG via orogastric gavage for 28 days, 6 days per week. Plant residues at 500 mg/kg were suspended in 1% CMC. The first group was assigned as the control and received distilled water, whereas group II (MSG-treated) received 900 mL of MSG. The remaining 4 groups (groups III to VI) received MSG and the respective plant extract at a dose of 500 mg/kg (Table 5).

MSG was administered orally for 28 days to induce uterine fibroids, followed by 30 days of treatment with the respective plant extracts. After the 30-day treatment period and twenty-four hours following the last dose, the rats were sacrificed under light ether anesthesia. Samples of blood were obtained by cardiac puncture from all animals for hormonal analysis (Figure 7). The uteri of four animals per group were dissected out and cleaned of adherent fat, and then fixed in 10% formalin saline. Animals were euthanized between 9:00 AM and 11:00 AM to minimize circadian variation. Estrous cycle phase was not determined prior to sacrifice, and this is acknowledged as a potential source of biological variability in hormone and uterine morphology outcomes. Paraffin-embedded tissue blocks were prepared from the four rats’ uteri. From each block, four stained slides were prepared, each containing three sections. Slides were examined blindly by three independent observers. Only observations with full agreement among the observers were included in the results.

**Table 5:** Experimental study design.

No.	Groups	Intervention	Treatments
I	Control	DW	0.5 mL distilled water
II	MSG-treated	900 mg/kg/day	0.5 mL MSG
III	*S. argel*	900 mg/kg/day	500 mg/kg *S. argel* extract in CMC
IV	*S. palustris*	900 mg/kg/day	500 mg/kg *S. palustris* extract in CMC
V	*P. armeniaca*	900 mg/kg/day	500 mg/kg *P. armeniaca* extract in CMC
VI	*L. sativum*	900 mg/kg/day	500 mg/kg *L. sativum* extract in CMC

Treatments and interventions were administered via intragastric oral gavage. DW = distilled water, MSG = monosodium glutamate.

### 4.5. Biochemical, Hormonal and Histological Analyses

#### 4.5.1. Assessment of Antioxidant Status

ELISA kits were supplied by Neo-Science Equipment & Chemicals Trading LLC (Neo-Science Equipment & Chemicals Trading LLC, Abu Dhabi, UAE. Website: www.neoscience.ae (accessed on 17 March 2026), TL No. CN-1767551, P.O. Box: 232672, Abu Dhabi—UAE, Tel: +971-45850861). 

Catalase activity was determined spectrophotometrically using a commercial catalase assay kit from Sigma-Aldrich (Sigma-Aldrich, St. Louis, MO, USA). The assay measures the decomposition of hydrogen peroxide, with the decrease in absorbance being proportional to catalase activity. 

SOD activity was measured using a spectrophotometric assay kit, which is based on the inhibition of the reduction of nitroblue tetrazolium (NBT) by superoxide radicals. Absorbance was recorded at 560 nm. 

Glutathione levels were quantified using the competitive inhibition enzyme immunoassay technique (ELISA kit) following the manufacturer’s protocol (Abclonal, RK04298, Abclonal, Wuhan, China; RK04298). Tissue homogenates were prepared, and the samples were incubated with biotinylated GSH, and the absorbance was measured at 450 nm to determine GSH concentration.

#### 4.5.2. Assessment of Hormonal Profiles

Estrogen levels were measured using an ELISA kit (Abclonal, RK00651) based on a competitive inhibition reaction between biotin-labeled E2 and unlabeled E2 from the sample. The intensity of the developed color was inversely proportional to the concentration of E2 in the sample. The assay was conducted in accordance with the kit’s instructions, with absorbance measured at 450 nm. The calibration range for estrogen ELISA was 25–200 pg/mL; for ER-α: 0.2–5 ng/mL

Progesterone levels in serum were measured using a specific ELISA kit (Abclonal, RK09247). The serum samples were prepared, incubated with the assay reagents, and the absorbance was recorded at 450 nm.

ER levels were determined using a specific ELISA kit (Abclonal, RK07561), which selectively detects estrogen receptor alpha (ER-α). The assay is based on a sandwich enzyme immunoassay technique, with the absorbance recorded at 450 nm. All procedures were carried out according to the manufacturer’s instructions.

#### 4.5.3. Histological Analysis

Uterine tissues were excised, cleaned of adhering fat, and fixed in 10% neutral buffered formalin solution (pH 7.4). The samples were dehydrated in graded alcohols, embedded in paraffin, and sectioned at 5 µm thickness. Sections were stained with hematoxylin and eosin (H&E) for general histological evaluation and Masson’s trichrome for collagen fiber and smooth muscle visualization. Microscopic analysis focused on endometrial epithelial integrity, myometrial thickness, glandular architecture, collagen deposition, and inflammatory infiltration. The slides were examined using a microscope (Olympus CX21, Tokyo, Japan) connected with a camera (Olympus-DP20, Olympus U-TV0.5X C-3-SN 8M 05614-T2 Tokyo, Japan-Made in the Philippines). Paraffin-embedded tissue blocks were prepared from three rats per group. From each block, four stained slides were prepared, each containing three sections. Slides were examined in a blind manner by three independent observers. Only observations with full agreement among the observers were included in the results [26].

Image analysis was performed using the color deconvolution tool of Aperio ImageScope software 12 (Leica Microsystems, Wetzlar, Germany) to quantify collagen in Masson trichrome-stained sections. Color deconvolution (color separation) was performed to isolate and measure the area of only the green color of collagen fibers and calculate the percentage of that area relative to the total area of a high-power field (HPF) photomicrograph. Also, the thickness of the myometrium was measured in different groups.

#### 4.5.4. Statistical Analysis

Statistical analysis was performed using GraphPad Prism version 10.3.1 (GraphPad Software, San Diego, CA, USA). All data are expressed as mean ± standard deviation (SD). Differences between groups were assessed using one-way analysis of variance (ANOVA) followed by Tukey’s multiple comparisons post hoc test to identify specific group differences. A *p*-value of <0.05 was considered statistically significant. The number of animals per group was *n* = 6, unless otherwise stated. Individual data points were plotted alongside bars to illustrate variability. No predefined inclusion or exclusion criteria were established. All animals completing the protocol were included in the final analysis.

## 5. Conclusions

In conclusion, this study explores preclinical evidence that *Lepidium sativum* and *Stachys palustris* possess promising therapeutic activity in mitigating MSG-induced uterine fibroid-like changes through their dual action as antioxidants and modulators of estrogen pathways. These findings suggest that select plant-based interventions could offer non-invasive, fertility-preserving alternatives to conventional fibroid therapies. Although *Prunus armeniaca* and *Solenostemma argel* exhibited milder effects, their distinct antioxidant and receptor-modulating actions warrant deeper mechanistic investigation. Future in vivo and clinical studies are needed to validate these results and potentially translate them into safe, effective, and affordable treatments for women affected by uterine fibroids.

## Figures and Tables

**Table 1:** Evaluation of the antioxidant effects of different plant extracts on the expression of estrogen receptors, estrogen level, and progesterone level.

Groups	Estrogen Receptors	Estrogen	Progesterone
Control	2.6 ± 1.2	105.7 ± 28.2	2.4 ± 1.5
MSG	3.6 ± 0.4	154.3 ± 13.6	2.0 ± 1.1
*S. argel*	1.8 ± 0.9	131.8 ± 17.6	3.6 ± 3.5
*S. palustris*	2.9 ± 0.4	129.7 ± 6.5	3.0 ± 0.8
*L. sativum*	1.8 ± 0.4	137.0 ± 15.7	3.4 ± 1.7
*Prunus armeniaca*	2.6 ± 1.6	130.4 ± 16.8	2.8 ± 1.5

Note: Statistically significant differences were observed in ER-α expression between MSG and *S. argel* or *L. sativum* (*p* < 0.05), and in estrogen levels between MSG and control (*p* < 0.01). No significant differences were found in progesterone levels across the group.

**Table 2:** Effect of different herbal extracts on the microscopic structure of the uterus in MSG-induced uterine alterations in Wistar rats.

Rats Treated with	Endometrial Epithelium	Endometrial Glands	Endometrium Thickness	Mononuclear Cellular Infiltration	Myometrium Thickness
DW only	Normal and intact	Normal	Normal	Normal	Normal
MSG only	Hypertrophied Hyperplastic with apoptotic cells seen and detached in some rats	Numerous Irregularly dilated and distorted	Markedly thickened	Marked infiltration mainly with eosinophils	Markedly thickened and more vascular
*L. sativum* after MSG	Intact and normal	Normal in number and structure	Normal	Normal	Mild thickening
*S. Palustris* after MSG	Intact and normal	Hyperplastic but of normal structure	Normal	Normal	Mild thickening and more vascular
*S. argel* after MSG	Slightly hypertrophied	Normal	Hypertrophied	Normal	Moderate thickening
*P. armeniaca* after MSG	Hypertrophied	Numerous but normal structure	Normal	Mild infiltration	Moderate thickening

**Table 3:** The score of different histological changes in the different experimental groups.

Histological Changes/Treated Groups	DW	MSG	*L. sativum* after MSG	*S. Palustris* after MSG	*S. argel* after MSG	*P. armeniaca* after MSG
Endometrial epithelium	0	3	0	0	2	2
Endometrial glands	0	3	0	2	0	1
Endometrium thickness	0	3	0	1	2	1
Mononuclear cellular infiltration	0	3	0	0	0	1
Myometrium thickness	0	3	0	2	3	3

Normal = 0, mild = 1, moderate = 2, severe = 3.

**Table 4:** Mean area % of collagen deposition in different experimental groups.

Groups	Mean Area % of Collagen Deposition ± SD	Thickness of Myometrium
Control	7.71 ± 0.64	45 ± 3.35
MSG	18.47 ± 0.35	112.5 ± 8.73
*S. argel*	14.57 ± 1.02	80 ± 6.42
*S. palustris*	22 ± 0.78	50 ± 5.17
*L. sativum*	13.15 ± 0.96	47.5 ± 4.09
*P. armeniaca*	15.50 ± 0.65	62.5 ± 2.19

## Data Availability

The original contributions presented in the study are included in the article; further inquiries can be directed to the corresponding author.

## List of Abbreviations

CMC	Carboxymethylcellulose
CAT	Catalase
CO.	Company
DPPH	2,2-diphenyl-1-picrylhydrazyl
DW	Distilled water
E2	Estrogen
ER-α	Estrogen receptor alpha
ECM	Extracellular matrix
GSH	Glutathione
GnRH	Gonadotropin-releasing hormone
g	Gram
H&E	Hematoxylin and eosin
HPF	High-power field
Kg	Kilogram
LD50	Lethal dose 50%
*L. sativum*	*Lepidium sativum*
L	Liter
μg	Microgram
mg	Milligram
mL	Milliliter
MSG	Monosodium glutamate
nm	Nanometer
ng	Nanogram
NBT	Nitroblue tetrazolium
NSAIDs	Non-steroidal anti-inflammatory drugs
pg/mL	Picograms per milliliter
*P. armeniaca*	*Prunus armeniaca*
QE	Quercetin equivalent
ROS	Reactive oxygen species
Rf	Retention factor
SD	Standard deviation
SPRMs	Selective progesterone receptor modulators
*S. argel*	*Solennostemma argel*
*S. palustris*	*Stachys palustris*
SOD	Superoxide dismutase
TLC	Thin layer chromatography
UV	Ultraviolet
UV-Vis	Ultraviolet–visible
UFs	Uterine fibroids
Ut.G	Uterine glands
